# Clearance of hepatitis C virus is associated with early and potent but narrowly-directed, Envelope-specific antibodies

**DOI:** 10.1038/s41598-019-49454-w

**Published:** 2019-09-16

**Authors:** Melanie R. Walker, Preston Leung, Auda A. Eltahla, Alexander Underwood, Arunasingam Abayasingam, Nicholas A. Brasher, Hui Li, Bing-Ru Wu, Lisa Maher, Fabio Luciani, Andrew R. Lloyd, Rowena A. Bull

**Affiliations:** 1 Viral Immunology Systems Program, The Kirby Institute, Sydney, Australia; 2 School of Medical Sciences, Faculty of Medicine, The University of New South Wales, Sydney, Australia

**Keywords:** Hepatitis C virus, Outcomes research

## Abstract

Hepatitis C virus (HCV) is one of very few viruses that are either naturally cleared, or alternatively persist to cause chronic disease. Viral diversity and escape, as well as host adaptive immune factors, are believed to control the outcome. To date, there is limited understanding of the critical, early host-pathogen interactions. The asymptomatic nature of early HCV infection generally prevents identification of the transmitted/founder (T/F) virus, and thus the study of host responses directed against the autologous T/F strain. In this study, 14 rare subjects identified from very early in infection (4–45 days) with varied disease outcomes (n = 7 clearers) were examined in regard to the timing, breadth, and magnitude of the neutralizing antibody (nAb) response, as well as evolution of the T/F strain. Clearance was associated with earlier onset and more potent nAb responses appearing at a mean of 71 days post-infection (DPI), but these responses were narrowly directed against the autologous T/F virus or closely related variants. In contrast, a delayed onset of nAbs (mean 425 DPI) was observed in chronic progressors that appear to have targeted longitudinal variants rather than the T/F strain. The nAb responses in the chronic progressors mapped to known CD81 binding epitopes, and were associated with rapid emergence of new viral variants with reduced CD81 binding. We propose that the prolonged period of viremia in the absence of nAbs in these subjects was associated with an increase in viral diversity, affording the virus greater options to escape nAb pressure once it emerged. These findings indicate that timing of the nAb response is essential for clearance. Further investigation of the specificities of the early nAbs and the factors regulating early induction of protective nAbs is needed.

## Introduction

Hepatitis C virus (HCV) is a major cause of chronic liver disease globally^1,2^. Following acute infection, approximately 75% of people fail to clear the virus, resulting in chronic hepatitis^3^ with progressive hepatic fibrosis and ultimately cirrhosis, liver failure and an increased risk of hepatocellular carcinoma^4,5^. Although the advent of direct acting antiviral (DAA) therapies offer great promise for disease control, many challenges remain in global elimination, including the need to develop a prophylactic HCV vaccine, which is likely to be an essential component of the prevention strategy^6^. The development of a successful vaccine has been hindered firstly by our limited understanding of what constitutes a protective immune response, and also the considerable challenge posed by the vast heterogeneity of the virus, both across populations and within individual hosts, due to the highly error prone HCV replication^7^. The current lead candidate is a T cell based vaccine which recently completed a Phase II study which proved ineffective in preventing chronic infection^8^. Nevertheless, it is likely that a successful HCV vaccine will need to induce both T and B cell immune responses, as existing data suggests both are associated with clearance^9–11^.

Transmission of HCV is associated with a strong genetic bottleneck, with one or very few transmitted/founder (T/F) viruses establishing infection upon blood-to-blood transmission despite an innumerable number of individual variants in the source^12–14^. This is followed by a second genetic bottleneck at ~100 days post infection, when T/F viruses are cleared, but are replaced in a selective sweep by new variants in chronic progressors, which carry mutations in CD8 T-cell and B-cell epitopes^13^. The role of neutralizing antibodies (nAbs) in clearance has previously been controversial with early studies indicating that such responses were not associated with clearance^15–17^, but more recent definitive studies clearly implicate nAbs^9,11,18^. The exact timing of these nAb responses and their relation to viral evolution is still poorly characterized, as it is now established that the viral quasispecies will have already evolved from the original T/F within 100 days post infection, this raises the question as to whether the earliest responses specifically target the T/F virus^9,10,16,19^. Only one small study with two subjects has been reported and both developed broadly neutralizing nAbs (BnAbs)^20^. However, both subjects were likely to be non-representative as one took almost a year to clear infection (six months is taken as the typical cut off for primary infection), and the other was co-infected with two HCV strains, and both longer duration and co-infection have been shown to be associated with an increased breadth of the nAb response^21^. Therefore, the examination of very early responses targeting the T/F viruses in a well-defined cohort is still lacking.

HCV cell entry is a multistep process that involves dimerized Envelope (E) 1 and E2 present on the surface of the virion. E1E2, along with low-density lipoproteins (LDL), dock to several receptors, including, cluster of differentiation 81 (CD81), scavenger receptor class B member 1 (SR-B1), claudin-1 (CLDN1) and occludin (OCLN) (74–81), on hepatocytes and enter the cell via clathrin-mediated endocytosis (71–73). The majority of the BnAbs characterized to-date (AR1, AR3, Domain C, Domain D, Domain E) act via blocking the interaction between E1E2 and CD81^22–30^.

There is reasonable evidence from liver transplant studies to indicate that T/F variants have higher infectivity^31^, which may be via more efficient use of CD81. It is still unknown if differences in CD81 usage by the T/F variants could contribute to different clinical outcomes. There is accumulating evidence from *in vitro* studies that modulation of host receptor usage (e.g. reduced CD81 or SRBI binding) is an important mechanism of immune escape, as the nAbs then have no effective role in blocking ongoing replication^32^. This has also been supported by *in vivo* data from two small studies, each with two HCV infected patients analyzed longitudinally, which indicated that reduced CD81 receptor binding coincided with emergence of nAbs^20,33^. However, all three clearance outcomes documented in these studies were unusually delayed beyond 6 months post-infection.

In this study, longitudinally collected samples from two rare cohorts of very recently infected individuals followed to either natural clearance or chronic infection outcomes were examined. The properties of the nAb responses and the viral traits were examined over the course of infection and in relation to infection outcome.

## Results

### Generation of infectious autologous T/F viruses from subjects with varied disease outcomes

Longitudinally collected samples from 14 newly viremic, but still seronegative, subjects were selected for this study. The estimated days post infection (DPI) of the initial infection time point ranged from 4 to 45 days (median 30). Of the 14 subjects, seven naturally cleared the primary infection (termed here clearers) and seven developed chronic infection (termed here chronic progressors) (Table 1). The median time to natural clearance was 178 DPI (range 124–487).

**Table 1:** Subject characteristics and time point analysis.

Subject ID^a^	Age at infection	Sex	Disease outcome	GT^b^	First sampling point (DPI^c^)	Time to clearance	E2 seroconversion point (DPI^n^)	Initial viral load	No. of samples sequenced^d^	Number of T/F^e^ viruses	Infectious in HCVpp assay	Fold over background
168_Cl	24	M	Clearer	1b	4	142	38	10989916	3	1	Yes	316.2
277_Cl	25	M	Clearer	3a	39	195	69	5482503	3	1	No	0.2
306_Cl	24	F	Clearer	1a/2b	5	487	40	8462679	3	1	No	1.8
360_Cl	29	M	Clearer	3a	30	178	44	5648631	3	1	Yes	8.9
4032_Cl	22	M	Clearer	3a	44	124		237930	2	1	Yes	10.7
4087_Cl	32	F	Clearer	1b	45	139	66	13118082	2	1	No	5.3
686_Cl	23	F	Clearer	1a	33	316	33	287770	4	1	Yes	26.9
023_Ch	22	M	Chronic	1a	36		51	19234348	6	2	Yes	16.4^g^, 0.7^h^
240_Ch	21	M	Chronic	3a	44		265	54887	5	1	No	4.3
256_Ch	31	M	Chronic	1a	44		69	34149824	3	1–10 (unresolved)	Yes	7.2^i^, 6.5^j^, 0.2^k^
4059_Ch	31	M	Chronic	1a/2b	30		69	3676682	1	1	N/A^f^	N/A
HOK_Ch	26	F	Chronic	1b	30		77	733849	4	1	Yes	0.2^l^, 9^m^
THD_Ch	25	M	Chronic	1a	16		51	235662	4	1	Yes	67
THG_Ch	28	M	Chronic	1a	2		282	140200	3	1	Yes	67

^a^Identification, ^b^Genotype, ^c^Days post infection, ^d^ Next generation sequencing, ^e^Transmitted/Founder, ^f^Not available, ^g^T/F1, ^h^T/F2, ^i^T/F1, ^j^T/F2, ^k^T/F3, ^l^T/F, ^m^30DPI, ^n^Measured as the midpoint between last negative and first positive timepoint.

The T/F viruses were estimated from the distribution of variants at the earliest sampling time point as previously described^13,34–38^. To determine the number of T/F viruses that established infection, Poison Fitter and phylogenetic analyses were performed on the haplotypes generated across the Core-E1E2 region to determine if there was 1 or >1 T/F virus (Supplementary Fig. 1). Twelve of the 14 subjects had a single T/F virus, and the E1E2 was cloned for all these variants (Table 1). Subject 023_Ch (the_Ch suffix refers to the chronic infection outcome for this subject), as previously published, had two T/F viruses identified^13^, and subject 256_Ch, had an unresolved number of variants, estimated to be between 1 and 10 (Supplementary Fig. 1). Both T/F E1E2 viruses were cloned for subject 023_Ch, and subject 256_Ch had three E1E2 variants selected for cloning, with the most frequent haplotype selected from each of the three main phylogenetic clusters. In total, 16 T/F variants were considered for HCVpp production.

The 16 T/F HCVpp generated from the 14 subjects were tested for infectivity. A total of 9 out of 16 (56%) T/F HCVpp were infectious *in vitro*, defined as greater than five-fold over background (Supplementary Fig. 2). Those that were non-infectious, included three T/F HCVpp from seven clearer T/F viruses (43%; non-infectious clones from 306_Cl, 277_Cl and 4087_Cl) and four T/F HCVpp from nine chronic progressor T/F viruses (44%; non-infectious clones from 240_Ch T/F, 023_Ch T/F2, HOK T/F and 256_Ch V3). Correctly folded E1E2 was confirmed for all T/F HCVpp by binding with a panel of 12 well-characterized mAbs, with the exception of 277_Cl and HOK_Ch (Supplementary Table 1). For HOK_Ch, an infectious variant from a sample collected 30 DPI was used for HCVpp, which only differed from the T/F at residue R446K (Supplementary Fig. 8).

### Clearers have an earlier and potent nAb response

In order to determine if the timing of nAb development against the autologous T/F virus(es) was associated with disease outcome, nAb activity in plasma was tested longitudinally against the autologous T/F HCVpp. For the five subjects with non-infectious HCVpp (277_Cl, 240_Ch, 306_Cl, 4087_Cl and 4059_Ch), representative HCVpps were chosen based on sequence similarity (Supplementary Table 2). All samples were also tested for neutralization against control pseudo-particle VSV-G to confirm specificity.

**Figure 1:**
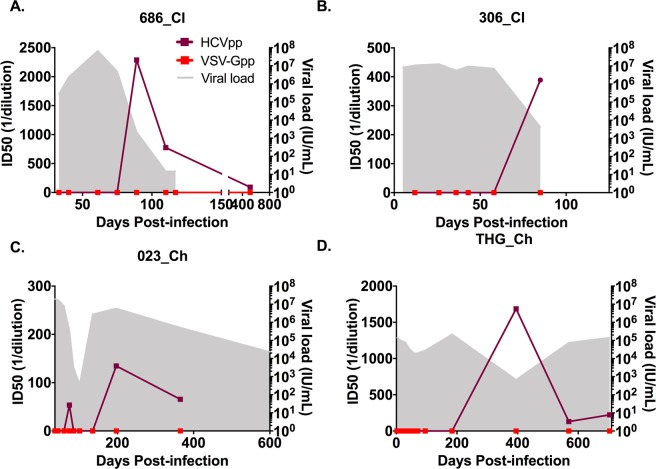
HCV and VSV-G neutralizing activity and HCV RNA levels (IU/ml) were examined longitudinally for clearers and chronic progressors. Panels(A,B) show representative subjects who cleared the infection. Panels (C,D) show representative subjects who developed chronic HCV infection. The shaded area represents the longitudinal HCV RNA levels (IU/ml). The maroon line represents neutralising antibody (nAb) ID50 titer with squares representing time points tested on autologous virus and circles representing time points tested on heterologous virus. The red line represents neutralization of control pseudo-particle VSV-G. Neutralization results were generated from quadruplicates using two-fold dilutions from 1/40 to 1/2560.

All 14 subjects demonstrated HCV-specific neutralizing activity, as indicated by a 50% neutralization capacity at a 1/40 dilution for at least one-time point during infection (Fig. 1 and Supplementary Figs 3 and 4). No nAb activity against VSV-G was detected in any subject or sample (Fig. 1).

**Figure 2:**
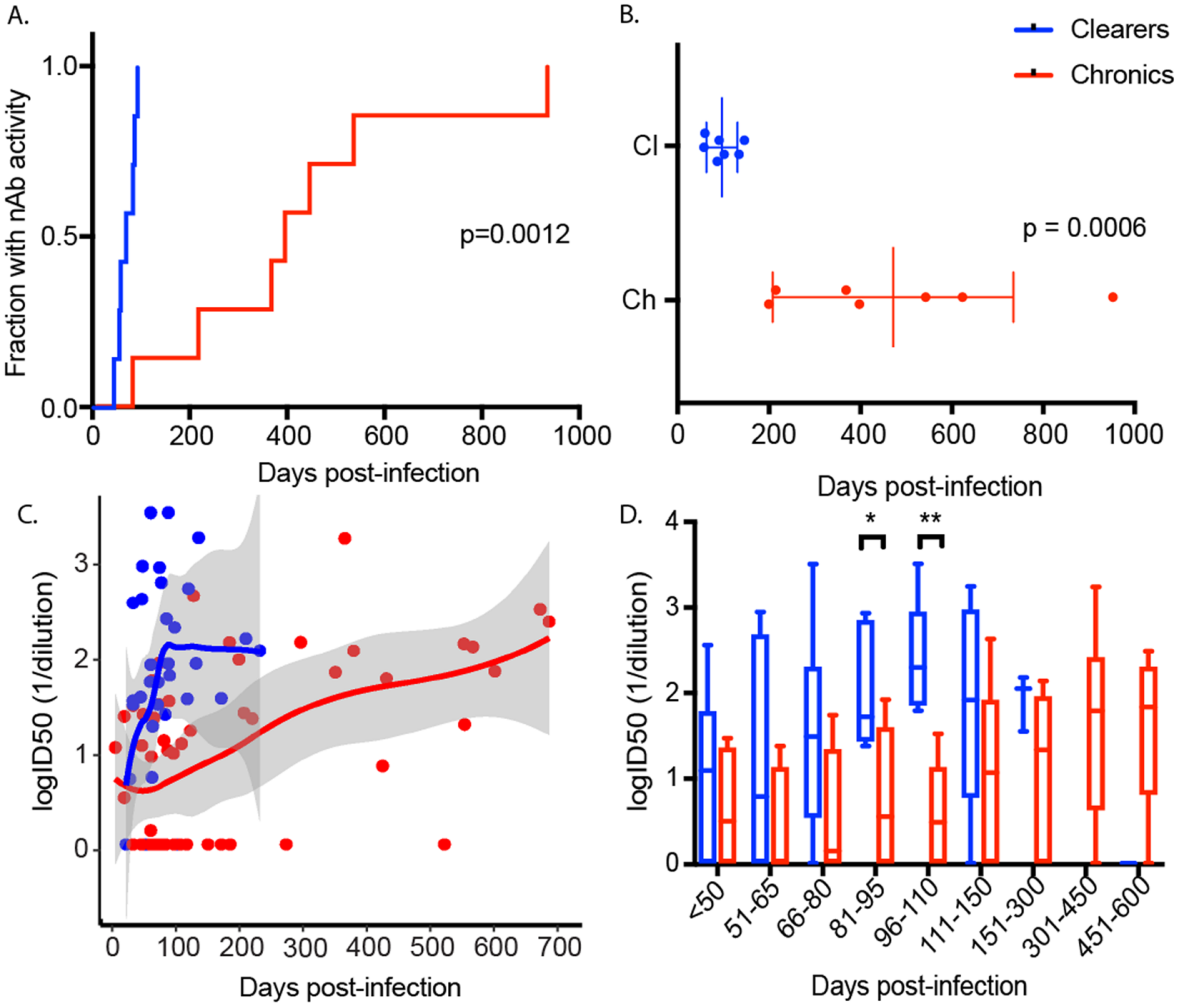
Timing and potency of nAb responses in clearers and chronic progressors. (**A**) Kaplan-Meier survival analysis was performed to compare the timing of the first nAb responses, defined here as a 50% reduction of HCVpp infectivity at the highest serum concentration (i.e. lowest dilution, 1/40) between clearers (blue) and chronic progressors (red). Clearers had significantly earlier nAb responses with a median of 71 days post-infection (DPI) (range 44–95) compared to a median of 425 DPI in chronic progressors (range 74–945) (**B**). The timepoint where the peak ID50 responses recorded was significantly earlier in clearers (**C**). The logID50 values were stratified into time windows, with each subject only represented once per time window, and curves were fitted with loess regression (grey shading represents 90% CI) for clearers (blue) and chronic (red) subjects. (**D**) A repeated measures ANOVA was applied to the nAb responses groupwise, and then ‘protected’ timepoint comparisons until 300 DPI. The logID50 values changed significantly over time F(2.39–14.29) = 6.88, p = 0.006) and differed by outcome group with significantly higher ID50 values in clearers than chronic progressors between 81–110 DPI (*p = 0.05, **p = 0.005).

Analysis of the longitudinal data indicated that HCV-specific nAbs first emerged (defined as an ID50 < 1/40) significantly earlier in clearer subjects at a median of 71 DPI (range 44–95) compared to a median of 425 DPI in chronic progressors (range 74–945, p = 0.0012, Fig. 2A). The timing of the peak in magnitude of the nAb response was also earlier in clearer subjects at a median of 89 DPI (range 56–144) when compared to chronic progressors, who had a median of 538 DPI (range 197–945, p = 0.0006, Fig. 2B). In order to examine if the earlier nAb responses were more potent in the clearers, the data was stratified into time windows ( < 50, 51–65, 66–80, 81–95, 96–110, 111–150, 151–300, 301–450, 451–600). Each subject was only represented once per time window with the largest value selected per window. Loess regression analysis of the inhibitory-dose (ID)50 data partitioned into time windows illustrated rapid emergence of highly potent nAbs in clearers (Fig. 2C). A repeated measures ANOVA was applied for groupwise, and then ‘protected’ timepoint comparisons until 300 DPI. The logID50 values changed significantly over time F(2.39–14.29) = 6.88, p = 0.006) and differed by outcome group with significantly higher ID50 values in clearers than chronic progressors between 81 and 110 DPI (81–95 window p = 0.024, 96–110 window p = 0.002) (Fig. 2D).

### Early nAb responses are narrowly directed

**Figure 3:**
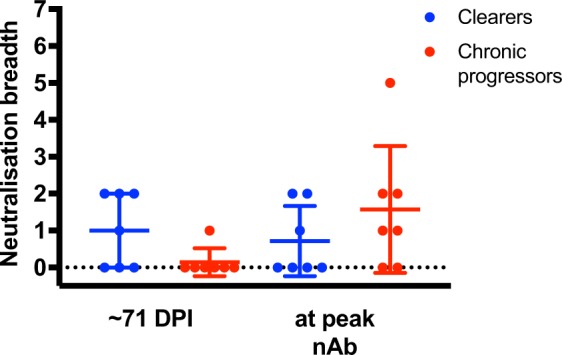
Neutralization breadth of clearers and chronic progressors. nAb breadth was calculated longitudinally for both clearers and chronic progressors. All samples were tested against HCVpp subtypes: 1a, 1b, 2a, 2b, 3a, 4a and 6a. The number of HCVpp neutralized by each sample was calculated and compared. Comparisons using a Wilcoxon rank test were made between clearers and chronic progressor samples collected at timepoint matched samples closest to 71 DPI (median time to nAb emergence in clearers), and also at the peak nAb response.

In order to confirm that the nAb activity was truly delayed in the chronic progressors and not influenced by neutralization sensitivity of the T/F, plasma samples from each subject collected closest in time to 71 DPI (the median time to nAb emergence in clearers) was examined for nAb activity against a panel of 7 HCVpp bearing E1E2 representing seven subtypes (1a, 1b, 2a, 2b, 3a, 4a and 6a) (Fig. 3, and Supplementary Fig. S5). In the samples from the 7 chronic progressors only one had weak neutralization of one of the HCVpp at 51%. For comparison, plasma from four of the 7 clearers demonstrated neutralization of one or more HCVpp from the panel, but the nAb activity was still narrow, with a maximum of only two HCVpp neutralized per subject which was not significantly different to breadth of the delayed onset responses in the chronic progressors (p = 0.1189).

The breadth of neutralization at the time of the peak ID50 response detected longitudinally was also compared between the infection outcome groups, with a non-significant trend towards greater breadth in chronic progressors (p = 0.3998). It should be noted that sample selection for this analysis was not time matched as the nAb responses in chronic progressors emerged much later (clearers range: 58–144 DPI, chronics range: 197–945 DPI) than clearers, and likely accounts for the previously described increase in chronically infected subjects^21^. It was interesting to note that of the 8 subjects with neutralization of at least 1 of the HCVpp panel, 7 subjects only neutralized viruses that were of the same genotype as their infection. In combination, these data indicate that early nAb responses are narrowly directed.

As there are instances in HIV infection where the delayed nAbs are specific for variants which emerge longitudinally, rather than the T/F variant, this was tested for two subjects, THD_Ch and THG_Ch, who shared the same T/F (believed to have been infected from the same source) and had three longitudinal variants with good infectivity in the HCVpp system. The variants, THD_72DPI and THG_58DPI and THG_184DPI (named for the timepoint of isolation) were all the dominant variant at that timepoint. The HCVpp for THD were tested against plasma collected across five time points (85, 109, 198, 394, 583 DPI) and the HCVpp for THG were tested across two time points (184 and 380 DPI) with plasma at a 1/40 dilution (Supplementary Fig. S6). Interestingly, responses against the THG_58DPI variant revealed a neutralization profile identical to those against the T/F variant in plasma from both of the timepoints tested. The other two variants, THD_72DPI and THG_184DPI, were more sensitive to neutralization than the T/F variant with plasma from all timepoints tested, but the 1/40 dilution was only neutralizing at greater than 50% (the defined cutoff for nAb activity) at the same time point as the T/F variant (394 and 380 DPI for THD and THG, respectively). This finding, along with the delayed E2 seroconversion (Table 1), supports the observation that nAb responses were delayed in these two chronic progressors, and raises the possibility that the nAb response may have been induced by the later variant which then became cross-reactive against the T/F variant as the response broadened, rather than being induced by the T/F. It is also possible T/F-induced responses developed earlier but were below the limit of detection of these assay systems.

### nAb activity is delayed in chronic progressors until multiple known epitopes are targeted

Longitudinal epitope mapping was performed to determine which epitopes on the HCV Envelope proteins were being targeted, and therefore could be associated with nAb activity. Plasma samples that had a minimal signal-to-noise ratio (OD: cut-off ratio >1.8) in an E1E2 IgG ELISA were further characterized in a competition-based ELISA using the non-autologous genotype 1a H77 E1E2. A total of 43 plasma samples from 13 of the 14 subjects were characterized (the remaining subject, 4032_Cl, did not show binding to E1E2). MAbs targeting nine distinct antigenic regions (AR1, AR2, AR3/Domain B, AR4, AR5, Domain E, Domain D, Domain A and Domain C) were selected to determine which epitopes were being targeted longitudinally. A competition matrix was performed to ensure the epitope targeting by these mAbs could be distinguished from each other (Supplementary Fig. S9). Some crossover was apparent between Domain D (HC84.26) and AR3, and between AR2A and CBH7, but most epitopes could be distinguished.

**Figure 4:**
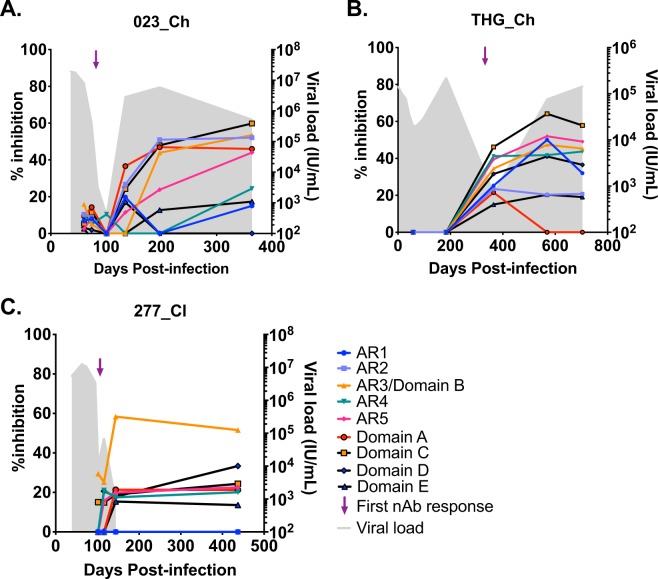
Epitopes targeted longitudinally in representative clearers and chronic progressors. Nine mAbs were used in competition binding assays to determine which epitopes (see key) were targeted throughout the infection. The maroon arrow indicates first nAb response (see key) and grey shading represents the viral load (see key). Representative chronic progressor subjects: 023_Ch (**A**) and THG_Ch (**B**), and clearer subject: 277_Cl (**C**), are shown. Responses in subjects 023_Ch, THG_Ch, and 277_Cl were mapped against non-autologous H77 E1E2.

In all seven chronic progressors, binding against multiple epitopes were identified in the competition binding assay in plasma samples taken as nAb activity emerged (Fig. 4 and Supplementary Fig. 7). In general, the amount of competition for the BnAbs could be seen to increase proportionately over time. However, in some subjects delayed emergence for some epitope binding was observed. Generally, nAb activity was delayed until high binding with multiple well-known neutralizing epitopes was evident. In summary, in chronic progressors the emergence of nAbs correlated with mapping to known nAb epitopes, and the number of epitopes targeted and the level of competition increased over time.

Surprisingly, limited competition in the binding assay with non-autologous E1E2 was observed in plasma samples collected from the clearers. Low competition, <30%, was observed for subjects 686_Cl, 306_Cl, 168_Cl and 4087_Cl (Supplementary Fig. 7). Subject 277_Cl, was the only clearer subject where competition was evident >30%, with an AR3-like dominant response (Fig. 4C). As most of the recognized HCV neutralizing mAbs act via inhibition of binding of HCV to CD81, a CD81 binding competition assay was also performed for the seven clearers, with only the plasma from the same subject, 277_Cl inhibiting CD81 binding (data not shown).

Due to the very narrow breadth of the binding activity in clearer samples, it was considered possible that lack of reactivity to the H77 E1E2 might have spuriously prevented competition against known nAb epitopes. Therefore, competition mapping against autologous E1E2 was attempted. However, for five of the seven clearers, despite good binding to rE2 in ELISA, poor binding to the lectin-bound autologous E1E2 was also observed (OD: cut-off ratio <1.8) (data not shown).

### Irrespective of infection outcome the majority of T/Fs utilize CD81, but as antibodies targeting well-characterized epitopes are generated, the E1E2-CD81 interaction is modulated

As HCV viremia continued in chronic progressors despite nAb activity, longitudinal sequence evolution from the T/F virus was sought in the next generation sequencing dataset of the longitudinally collected samples of clearers and chronic progressors. These data were examined for single amino acid polymorphisms (SAPs) occurring in >10% of the viral population within nAb epitopes, or CD81 binding sites (Supplementary Fig. 8). Limited viral evolution in antigenic regions was observed in clearers, with only two of seven subjects (4087_Cl and 306_Cl) having any SAPs identified.

In contrast, all seven chronic progressors had SAPs occurring in antigenic regions (Supplementary Fig. 8). A significant proportion of chronic progressors had SAPS occurring within AR3 (n = 7, p = 0.041), Domain B (n = 5, p = 0.041) and Epitope II (n = 6, p = 0.029) (Supplementary Table 3). Interestingly, mutations within these regions have been implicated in disruption of CD81 binding^25,27,39^. Four of the seven chronic progressors (023_Ch, 240_Ch, THD_Ch and THG_Ch) developed mutations in CD81 binding sites, equating to just over half of this group (57%). Notably, of these four subjects, all developed a mutation at residue Y443, which has been reported to be critical for E2 binding to CD81^39,40^. The viral population in all four subjects mutated away from the highly conserved tyrosine (Y), to a more polar, bulky, positively charged histidine (H) (THD_Ch, THG_Ch or 240_Ch), or to a bulky hydrophobic, isoleucine (I) (023_Ch).

To understand the functional impact that these mutations had on CD81 binding, HCVpp representative of the longitudinal chronic variants (n = 12) were produced, and a CD81 binding assay was performed on T/F variants from both clearers and chronic progressors, as well as the matched 12 longitudinal variants from chronic progressors. Correctly folded E1E2 for each HCVpp was confirmed with a panel of well-characterized mAbs (Supplementary Table 1). Table S1 indicated that at least within subjects the level of E1E2 detected via ELISA with a panel of mAbs was consistent across the longitudinal variants, though some variation between subjects was evident, likely a natural phenomenon of the neutralization profile. Unfortunately none of the well-characterised BnAbs were broad enough to enable proper quantification of the E1E2s.

**Figure 5:**
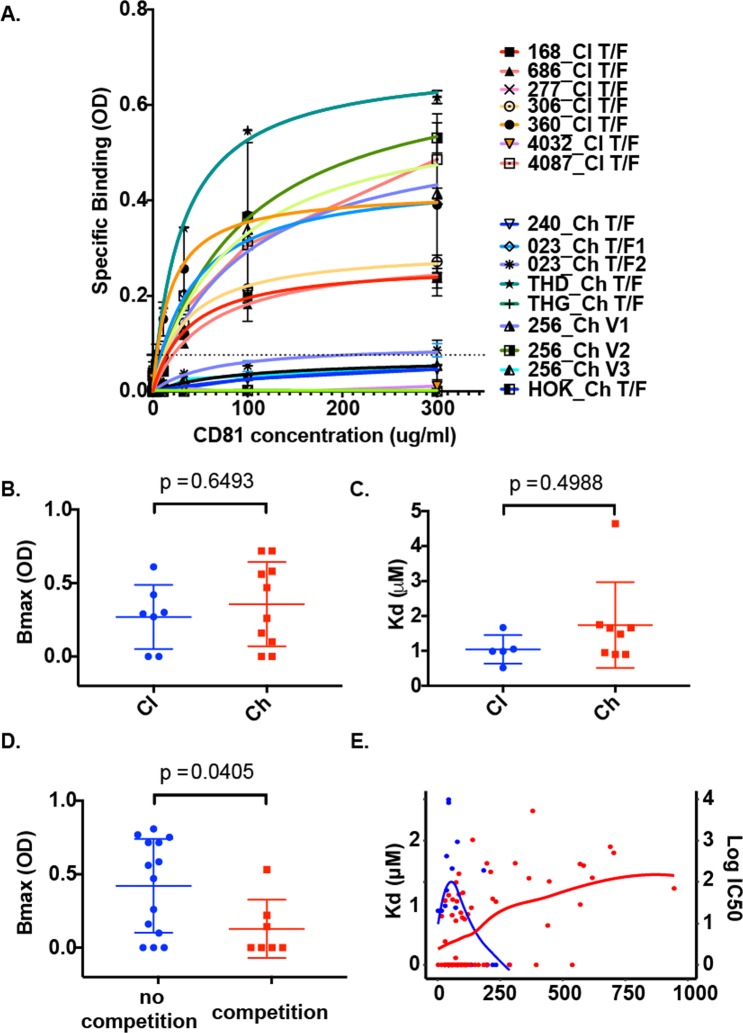
CD81 binding of viral variants by ELISA. Binding measurements were performed with HCVpp lysates incubated with a range of recombinant CD81 concentrations (0.4–300 μg/mL) (**A**). The Y-axis shows the mean OD values for bound CD81, and the dotted line represents background cut-off. Error bars represent SD. The saturation binding curves were fitted by non-linear regression. HCVpp incorporating E1E2 from T/F viruses from clearers (n = 7) are represented in warmer colors and from chronic progressors are represented in cooler colors (n = 9). All variants are labelled with subject number and clone identifier. Clearer and chronic progressor HCVpp T/F variants were compared for CD81 binding as *B*_max_ (**B**) and *K*_d_ (**C**). Variants were assessed for CD81 binding before and after emergence of anti-CD81 antibodies was observed using the epitope mapping ELISA (**D**). Loess regression curves illustrate the reciprocal relationship between the emergence of nAb activity (ID50) and the decline in *K*_d_ of the circulating viral variants over time from 6 of the subjects that developed chronic infection (**E**).

To determine whether CD81 binding of T/F variants influenced disease outcome or epitope recognition by nAbs, CD81 binding (*B*_max_) and affinity (K_d_) were compared between clearers and chronic progressors (Fig. 5). No significant difference was observed between the T/F variants of clearer and chronic progressor groups for *B*_max_ (Fig. 5B), or for K_d_ (affinity) (Fig. 5C). Examination of the later variants that emerged after nAb development in chronic infection revealed that both *B*_max_ and affinity to CD81 decreased in samples collected after the emergence of nAbs. The decrease in the CD81 *B*_max_, but not affinity, was significantly different in post-nAb variants, when compared to pre-nAb variants (p = 0.0405, Fig. 5D). Unfortunately, the lack of a broad binding mAb made it impossible to confirm that all E1E2 were added at comparable amounts, however as outlined in the methods, E1E2 was added to saturation and Table S1 indicates that at least within individual subjects, the variants had comparable mAb binding for the relevant mAbs. Loess regression curves were fitted for the CD81 Kd values from the representative variants isolated from the seven chronic progressors, in conjunction with the log ID50 scores (Fig. 5E) revealing the temporal trends towards decreased CD81 binding as the nAb ID50 increased.

### Higher diversity in viral population prior to nAb emergence predicts chronic progression

**Figure 6:**
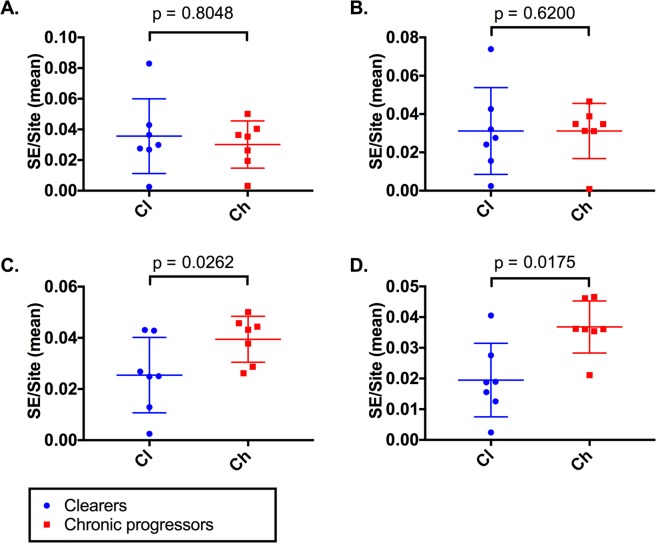
Shannon entropy across E1 and E2 regions. Shannon entropy (SE) across the E1 and E2 region was calculated and compared for clearers and chronic progressors over the first 51 days post-infection (DPI) (pre-Core/NS seroconversion) for both E1 (**A**) and E2 (**B**). Comparison of SE at the last sampling point prior to nAb emergence was also compared for clearers and chronic progressors across E1 (**C**) and E2 (**D**).

To test the hypothesis that delayed emergence of nAbs may advantage the virus by enabling a greater repertoire of emergent variants and hence potential for immune escape from nAb selection, the Shannon Entropy (SE) across the E1 and E2 regions was calculated from viral sequences and analyzed over the course of infection (Fig. 6). Firstly, in a time-matched comparison, the timepoint identified to have the greatest SE prior to 51 DPI was selected for each of the 15 subjects (Core/NS seroconversion in the commercial anti-HCV assay was defined as occurring at 51 DPI, see Methods for explanation). Comparison of the SE within E1 and E2 over this period showed no significant difference between clearers and chronic progressors (E1: clearers SE = 0.02989 chronic progressors SE = 0.03525, p = 0.8048. E2: clearers SE = 0.02758, chronic progressors SE = 0.03475, p = 0.6200) (Fig. 6A,B). In contrast, when the last time point prior to nAb emergence was compared, chronic progressors had significantly higher SE in both E1 and E2 when compared to clearers (E1: clearers SE = 0.02502, chronic progressors SE = 0.04318, p = 0.0262. E2: clearers SE = 0.01879, chronic progressors SE = 0.0361, p = 0.0175) (Fig. 6C,D).

## Discussion

Using a rare cohort of very recently infected individuals, this study assessed both virus and host characteristics longitudinally, seeking determinants of the clearance or chronic HCV infection outcomes. In terms of host characteristics, early development of autologous nAbs was associated with clearance. In chronic progressors, nAb production was delayed, which allowed a diverse viral quasispecies to emerge. Once nAbs did emerge in this group, the responses targeted epitopes known to be involved in CD81 binding, and rapidly drove a decrease in binding efficiency, likely as a result of the emergence of immune escape mutations.

Overall, the results reported here clarify some of the conjecture surrounding the role of nAbs in clearance. This study supports the findings of several nAb studies in primary HCV infection, that were performed with samples collected later in the course of infection and showed that early emergence of nAbs was associated with HCV clearance^9–11,41^. In addition, this study expands on the seminal paper in the single source German anti-D immunoglobulin cohort that showed that timing of nAb appearance was associated with disease outcome, but only examined a small number of samples collected before 100 days post-infection and three genotype 1 HCVpp – one being the T/F virus^10^. Here, we show similar early neutralization in clearers using 9 different T/F viruses, and early sampling points. The predominant use of autologous T/F variants in the neutralisation assays confirmed that nAbs in chronic progressors are truly delayed. In fact, for two subjects with very delayed nAb emergence and chronic infection outcome, the responses may have been induced by the later variants that replaced the T/F viruses, rather than toward the T/F virus itself. Further investigation of the mechanisms underpinning this delayed humoral response in chronic progressors is clearly warranted.

In a previous large primary infection study, it was suggested based on examination of neutralisation profiles against large panels of genotype 1 HCVpp, that infecting genotype did not influence NAb breadth and that clearers had broader nAb activity than chronic progressors^9,10^. We have extended these findings by testing with a truly broad panel of HCVpp (representatives of six genotypes), as well as the T/F virus HCVpp in many cases. We did not observe greater nAb activity against heterologous genotype HCVpp in clearers, indicating that the responses in this group are still narrowly directed to closely related viruses – noting that the smaller number of subjects limited the statistical power of the present study. In addition, the earlier sampling points in the present study, as five of the seven clearers in this study cleared the infection within the typical six month window, whereas in the previous study the subjects had a longer median time to clearance (275 days)^9^. This latter point is significant as it has been suggested that breadth continues to increase with ongoing infection^21^.

Concordant with the finding of limited breadth observed in clearers, minimal non-autologous competition with the panel of mapping mAbs targeting each of the well-recognized (and in some cases broadly neutralizing) E1E2 domains, was detected in clearer subjects. Likewise, the plasma from clearers was unable to compete for binding to CD81. In one clearer subject it was possible to perform E1E2 mAb competition mapping and low levels of competition against known mAbs was identified. In relation to the technical limitation of the inability to do competition mapping for the remaining clearer samples, we hypothesize that lectin immobilization of E1E2 may inhibit binding of some anti-E1E2 Abs as lectin will also target the highly glycosylated face of the Envelope. The mechanism behind this inhibition was not explored but could result from steric hindrance if the early Abs are pentameric IgM, or binding glycans directly, as has been shown in several studies in influenza and HIV infections^42^. The results presented here suggest that further examination of the characteristics of Abs in ‘rapid’ clearers is warranted. For example, the highly specific responses suggest HVR1 targeted antibodies may be contributing to the early nAb activity.

This study confirmed two previous studies which included a total of just four subjects, that T/F variants in primary infection have a higher dependency for CD81, and CD81 binding is reduced when anti-CD81 nAbs emerge^20,33^. This is noteworthy as nAbs that block CD81 binding are prevalent in chronic progressors, and appear ineffective in clearing infection^16,19,43,44^. This suggests that an alternate entry mechanism, such as an alternate receptor preference or cell-to-cell transmission may facilitate ongoing infection. In support of this notion, a recent *in vitro* study indicated that loss of SR-BI utilization was accompanied by increased affinity to CD81, suggesting some viruses may have preferential tropism for one receptor over the other^32^, as has been demonstrated in HIV^45^. However, a more recent study reported that the chronic variants replacing the T/Fs lose binding to both CD81 and SR-BI^20^. This concept requires further exploration, ideally with the HCV cell culture (cc) system, rather than the HCVpp system, as cell-to-cell transmission is thought to occur primarily through SR-B1 as well as the tight junction proteins, CLDN1 and OCLN, and following establishment of infection it is highly likely that the infection is propagated via cell-to-cell transmission^26,46–49^. This suggests that in addition to CD81 blocking antibodies, which are currently considered to be ideal for vaccine-induced protection^50^, a combination of antibodies which block interactions with multiple receptors may be required to limit avenues for escape. However, to-date few broad nAbs that block alternate entry mechanisms have been identified^51^.

The final finding from this study revealed that chronic progressors had significantly higher SE in both E1 and E2 when compared to clearers prior to nAb emergence. This implies that the reason new variants were able to emerge and replace the T/Fs in the chronic progressors was due to higher diversity at the time of nAb emergence. Interestingly, overtime E1 diversity had increased in the chronic progressors but E2 SE mean was comparable and it was the SE of E2 in the clearers that decreased suggesting that an additional pressure or perhaps dominance of a unique variant may be resulting in reduced diversity. We hypothesis that this reduced diversity in the clearers likely makes the population more susceptible to nAbs as RNA virus replication errors are a stochastic event, therefore having a greater repertoire of novel variants present is likely a critical factor in the emergence of escape variants.

In summary, the findings from this study suggest that a vaccine that can either rapidly induce high titre CD81 blocking antibodies in the plasma before the virus has time to generate sufficient diversity to escape, or can block entry via multiple mechanisms will be necessary for protection. This study also highlights the need to further characterise the epitopes involved in rapid clearance, and investigate the immunological mechanisms leading to delayed nAb responses in those who progress to chronic infection.

## Materials and Methods

### Subjects and samples

Samples were available from incident cases identified in two prospective cohorts of HCV-seronegative and aviremic, high-risk, individuals enrolled in the Hepatitis C Incidence and Transmission Studies (HITS) in prisons (HITS-p), or in the general community (HITS-c)^52–54^. In both cohorts, risk behavior data and blood samples were collected every six months to screen for seroconversion and for HCV RNA positivity. Early incident cases enrolled into a sub-cohort HITS-incident (HITS-i) when new onset viremia, but antibody negative status was recorded. These subjects were then sampled frequently for 12 weeks before antiviral treatment was offered and sometimes taken up after 24 weeks, thereby allowing designation of natural clearance or chronic infection outcomes. From the HITS-i cohort, longitudinally collected samples from 14 incident case subjects were used in this study. The date of infection was estimated by subtracting the average HCV pre-seroconversion window period, which has been estimated at 51 days^13,55–57^, from the midpoint between last seronegative and first seropositive time points.

### Ethics statement

Human research ethics approvals were obtained from Human Research Ethics Committees of Justice Health (reference number GEN 31/05), New South Wales Department of Corrective Services (05/0884), and the University of New South Wales (05094, 08081), all located in Sydney, Australia. Written informed consent was obtained from the participants. All methods were performed in accordance with the relevant guidelines and regulations.

### Virological assessments

In the prospective cohorts, sera were tested for HCV antibodies using the Abbott ARCHITECT anti-HCV chemiluminescent microparticle immunoassay (CIA) (Abbott Diagnostics). HCV RNA quantification was performed as described previously using either the VERSANT HCV RNA Qualitative Transcription Mediated Amplification (TMA) assay (Bayer Diagnostics) or the COBAS AmpliPrep/ COBAS TaqMan HCV assay (Roche)^58^.

### Viral sequencing and bioinformatic analysis

Near full length HCV genome amplification was performed using an nRT-PCR as described previously^13,59^. The next generation sequencing datasets of the full-length viral genomes at multiple time points during primary infection were largely published previously^13,34,59,60^. Briefly, both Roche 454 FLX (Basel, Switzerland) and Illumina (MiSeq Benchtop sequence, San Diego, USA) sequencing were performed on amplicons from longitudinal time points of the 14 subjects^59^. As described previously, a bioinformatics pipeline was used to clean and align reads generated from NGS^13,34^. To accurately detect single nucleotide polymorphisms (SNP) from the aligned and cleaned sequences, analysis was performed to correct for random technical errors using the software ShoRAH, LoFreq and Geneious^36,37^. Haplotype reconstruction was then performed on amino acids 1–800 of Core, E1, E2 and partial p7 of HCV poly-protein using ShoRAH^38^ to reconstruct viral variants within the quasispecies from the NGS datasets. Shannon Entropy was determined from the NGS data as previously described^13^. Consensus genomes are available on GenBank (Accession IDs to be added).

### Identification of HCV transmitted/founder and longitudinal variants

As described previously^13,34^, a statistical model, Poisson fitter, as well as phylogenetic analysis was applied to haplotypes to determine if the viral population had a star-like phylogeny, and whether the mutations observed followed a Poisson distribution, which signified infection with a single T/F virus^61^. Only HCV sequences obtained from the first available viremic time point for each subject were included in the founder virus analysis. PoissonFitter test was performed on reconstructed viral variants in E1/E2 region of the genome. If PoissonFitter did not reveal a star-like phylogeny, T/Fs were estimated based on tree conformation.

### Cell culture, antibodies and reagents

Human cell lines, Lenti-X^TM^ 293 T (Takara, Mountain View, CA, USA) and Huh7.5 (Apath, New York, NY, USA) were maintained at 37 °C and 5% atmospheric CO_2_ in growth medium containing High Glucose Dulbecco’s Modified Eagle Medium (HG-DMEM, Gibco, Thermo Fisher Scientific, Waltham, MA, USA) supplemented 10% v/v heat inactivated fetal bovine serum (FBS) (Gibco). Previously characterized HCV monoclonal antibodies (mAbs) AR1B, AR2A, AR3A, AR4A and AR5A were sourced from A/Prof. Mansun Law (Scripps, USA)^62^. CBH4G and CBH7 antibodies were purified from hybridomas purchased from ATCC (Manassas, VA, USA, PTA-4468 and PTA-4470). Antibodies HCV84.26 and HCV-1 were generated by transient transfection of plasmids provided by Prof Heidi Drummer (Burnet Institute, Australia), as previously described^63^. Antibodies were biotinylated using E2-Link Sulfo-NHS-LC-Biotin kit (Thermo Fisher Scientific).

CD81-large extracellular loop (LEL) glutathione S-transferase (GST) fusion protein in *E*. *coli* SURE competent cells was kindly provided by Prof. Shoshana Levy (Stanford University, USA). CD81-LEL was expressed and purified in SURE cells at the Protein Production Unit, Monash University, Melbourne, as described previously^64,65^.

Eleven E1E2 glycoproteins representing genotypes 1–6 (previously described^18,66–69^ and assigned GenBank accessions numbers: H77.20, AF011751; UKN1A20.8, EU155192; UKN1B12.16, AY734974; UKN1B5.23, AY734976; UKN2A1.2, AY734977; UKN2B2.8, AY734983; UKN2B1.1, AY734981; UKN3A1.9, AY734985; UKN3A13.6, AY894683; UKN4.11.1, AY734986; UKN6.5.340, AY736194; were kindly provided by Prof. Jonathon Ball and A/Prof. Alexander Tarr (University of Nottingham, United Kingdom). MLV gag/pol and luciferase plasmids were kindly provided by Prof. Francois-Loic Cosset (University of Lyon, France). A Vesicular Stomatitis Virus (VSV-G) Envelope plasmid was also provided by Prof. Stephen Foung (Stanford University, USA)^70,71^.

### HCVpp production, infection and neutralization

HCV pseudo-particles (HCVpp) were generated by co-transfection of Lenti-X 293 T cells with expression plasmids containing HCV Envelope proteins of interest, and the MLV gag/pol and luciferase vectors^65,72^. Culture supernatants containing HCVpp were harvested 48 hours post transfection and clarified of cellular debris by centrifugation. Cell pellets were stored at −80 °C until lysed. Neutralization assays were performed using infectious HCVpp that was at least five-fold over negative background (a pseudo-particle generated without HCV Envelope glycoproteins). Neutralization was tested on undiluted HCVpp if infectivity was less than 20 fold over negative background, or diluted to less than 20 fold over negative background with media^73^. For the neutralization assay, the pseudo-particle was diluted in heat inactivated (HI, 56 °C for 30 minutes) subject plasma and incubated for one hour at 37 °C, followed by the addition of polybrene at a final concentration of 4 μg/mL (Sigma-Aldrich, St Louis, MO, USA). This mixture was applied directly to Huh-7.5 hepatoma cells (Apath, L.L.C, New York, NY, USA), which had been seeded 24 hours earlier at 8 × 10^4^ on a 96 well opaque plate. Plates were then spun at 800 *g* for 2 hours and incubated for an additional 72 hours at 37 °C, 5% atmospheric CO_2_. To measure luciferase reporter expression, the BrightGlo luciferase assay reagent (Promega, Madison, WI, USA) was added at a 1:1 ratio directly to infected cells before luminescence (RLU) was measured (EnSpire Multimode, Perkin Elmer, Waltham, MA, USA). Positive neutralization controls included the HCV-specific mAbs, CBH-5 and HCV-1. Neutralization assays were performed in quadruplicate.

Neutralization of HCVpp was calculated using the formula: % inhibition = 1 − (inhibited activity)/(‘normal’ activity)] × 100, after subtraction of negative control (pseudo-particle generated without glycoproteins) RLU, where normal activity is HCVpp incubated with plasma from a healthy donor. Outliers of the quadruplicates (<2%) were excluded with a modified z-score method^74^. The 50% ID50 titer was calculated as the nAb concentration that caused a 50% reduction in RLU for each plasma/HCVpp combination tested in neutralization. All samples were also tested for neutralizing activity on control pseudo-particle VSV-G, to determine that nAbs were HCV E1E2 specific. All data were fitted using non-linear regression plots (GraphPad, Prism).

Cells and viral particles were lysed in radio immunoprecipitation assay buffer (RIPA, Sigma) with mammalian protease inhibitor as per manufacturer’s instructions (Sigma). After incubation on ice for 20 minutes, lysates were spun at 1000 g at 4 °C for five minutes to remove insoluble debris.

### Enzyme linked immunoassays

Microtiter plates, 96 well (Nunc Maxisorb, Thermo Fisher Scientific) were prepared with 500 ng of *Galanthus nivalis* (GNA) lectin, incubated for one hour, and then blocked for one hour. E1E2 lysate from transfected 293Tcells was added to a GNA lectin coated plate and incubated for 1 hour.

For epitope mapping, plates were prepared with H77 E1E2 as described above and blocked with bovine serum albumin (BSA). The bound E1E2 was then incubated with HI plasma at a final dilution of 1:10 for 1.5 hours. Only plasma samples with a binding signal to noise ratio > 1.8 were used (calculated as the healthy control + 2 SD). After test subject’s plasma was added to the bound antigen, nine biotinylated mAbs (AR1B, AR2A, AR3A, AR4A, AR5A, HCV-1, HCV84.26, CBH-4G and CBH-7) were added to wells at concentrations resulting in 70% binding. Binding of biotinylated mAbs was detected using HRP-conjugated streptavidin antibody. Percentage inhibition was calculated as (1 − Absorbance _test_/Absorbance _control_) × 100. The threshold for competition positivity was designated as the mean +2 SD of % inhibition from healthy control subjects (n = 14) and was calculated for each mAb; AR1 (8.6%), AR2 (22%), AR3/Domain B (17%), AR4 (9%), AR5 (11%), Domain A (20%), Domain C (11%), Domain E (8.6%) and Domain D (14%).

For CD81 binding, the bound antigens were incubated with purified CD81-LEL GST at dilutions of 0 μg/mL, 0.4 μg/mL, 1.2 μg/mL, 3.6 μg/mL, 11 μg/mL, 33 μg/mL, 100 μg/mL, and 300 μg/mL for one hour. The bound CD81 was then incubated in 10 μg/mL of CD81 antibody (1.3.3.22, Santa Cruz Biotechnology) for one hour after washing. Next, mouse anti-human IgG-Alkaline phosphatase (AP) conjugate antibody (1:5000, Thermo Fisher Scientific) was added for a further hour followed by a incubation with Working AP liquid substrate for ELISA (Sigma). Color development and absorbance were then measured at 405 nm. H77 HCVpp was used as a positive control and pseudo-particle generated without glycoproteins as a negative control, which was subtracted as background. CD81 assays were performed in intra-assay duplicates. The data were analysed by non-linear regression to measure the disassociation constant (K_d_) and the maximum binding level (*B*_max_), using Prism software (GraphPad).

To determine correctly folded pseudotyped E1E2, plates were prepared with GNA lectin as described above. Pseudotyped E1E2 lysates from transfected 293Tcells (described above) were quantified for total protein using a Bradford assay. Samples were diluted to contain equal amounts of protein and then diluted five times in two fold dilutions to determine saturation. After blocking with BSA, the bound E1E2 was incubated with CBH-5 at a final concentration of 1ug/mL. Binding of biotinylated mAbs was detected using HRP-conjugated streptavidin antibody. All E1E2 samples were found to be saturating at a 1:2 dilution (with the exception of 277 and HOK which did not bind to CBH-5). Plates were then prepared with GNA lectin and saturating amounts of HCVpp lysate as described above. Following blocking with BSA, the bound E1E2 was incubated with a panel of 12 well-characterized biotinylated mAbs (AR1B, AR2A, AR3A, AR4A, AR5A, HCV-1, HCV84.26, CBH-4G, CBH-7, CBH-5, A8 and mAb24) at a final concentration of 1 μg/mL for 1.5 hours. Binding of biotinylated mAbs was detected using HRP-conjugated streptavidin antibody. Signal to noise was calculated for all pseudotype E1E2 by dividing OD of pseudotyped E1E2 by OD of negative control (pseudo-particle generated without Envelope glycoproteins).

### Statistical analysis

All data analysis was performed and graphs created using GraphPad Prism Software (version 7.0, GraphPad) for Macintosh. Wilcoxon rank-sum test, Mann-Whitney test and Fisher’s Exact test (where appropriate) were used to evaluate statistically significant differences between groups. Loess curve fitting (local polynomial regression) was performed with the ggpot2 package in R (v3.3.1; The R Foundation for Statistical Computing) using the stat_smooth() function and default parameters. Statistical significance was defined as a p value less than 0.05. Results were expressed as mean ± standard deviation (SD).

Comparisons of plasma ID50 between the longitudinal samples of chronic progressors and clearers, across the study time points, were performed using a repeated measures ANOVA (SPSS). Comparisons between ID50 levels of clearers and chronic progressors across the study time points were then performed with protected t tests. GraphPad Prism Software was used for calculations and illustrations.

## Supplementary information

Supplementary Information
