# Supplementary material for: Clearance of hepatitis C virus is associated with early and potent but narrowly-directed, Envelope-specific antibodies
Source: Sci Rep. 2019 Sep 16;9:13300. doi: 10.1038/s41598-019-49454-w (PMC6746763; doi:10.1038/s41598-019-49454-w)
Supplement: Supplementary file 1 — Supplementary Information [file 41598_2019_49454_MOESM1_ESM.docx]

# Supplementary information

**Clearance of hepatitis C virus is associated with early and potent but narrowly-directed, Envelope-specific antibodies.**

Melanie R Walker^1, 2^, Preston Leung^1, 2^, Auda A. Eltahla^1, 2^, Alexander Underwood^1, 2^, Arunasingam Abayasingam^1, 2^, Nicholas A Brasher^1, 2^, Hui Li^1, 2^, Bing-Ru Wu^1, 2^, Lisa Maher^1^, Fabio Luciani^1, 2^, Andrew R. Lloyd^1^ and Rowena A. Bull^1, 2^.

*Viral Immunology Systems Program, The Kirby Institute****^1^*** *and School of Medical Sciences****^2^****, Faculty of Medicine, The University of New South Wales, Australia*

# Supplementary figures

**A.**

**B.**

**C.**

**D.**

**E.**

**F.**

**G.**

**H.**

**I.**

**J.**

**K.**

**L.**

**M.**

**N.**

Figure S 1 T/F virus analysis based on Core-E1E2 region of the viral genome. Phylogenetic analysis shows the genetic relatedness of viral variants at the first RNA-positive time point. The consensus sequence, labelled with subject number, represents the T/F. Haplotype sequences are labelled with “HAP” followed by haplotype prevalence from 1 onwards (HAP1, 2, 3 etc.), and a second number representing prevalence percentage in the viral population. The phylogenetic trees are consistent with an infection arising from one T/F virus as shown by the star-like phylogeny. If star like phylogeny was not observed, low frequency (<2.5%) haplotypes were excluded from the analysis due to likely difficulty identifying them during cloning. Star like phylogeny was then confirmed with a Poisson model. The phylogenetic trees of clearers 168_Cl, 277_Cl, 306_Cl, 360_Cl, 686_Cl, 4032_Cl and 4087_Cl are shown in panels A-G. Chronic progressors 023_Ch, 240_Ch, 256_Ch, 4059_Ch, HOK_Ch, THD_Ch and THG_Ch are shown in panels H-N. Phylogenetic trees were constructed using Mega with Maximum Likelihood methods using a GTR model of substitutions as suggested by model testing (73). *Note.* Amino acid sequence of HOK T/F and 306 T/F was the same as amino acid sequence of HAP1 (respectively), but varied slightly at synonymous sites.

Figure S 2 HCV T/F HCVpp infectivity. Transmitted/Founder (T/F) variants were selected in clearers (Cl) or chronic progressors (Ch) to assess neutralization. Results are expressed from three separate experiments in duplicate as relative light units. The dotted line represents functional HCVpp, which was calculated as five-fold over negative control (pseudo-particle generated without Envelope glycoproteins).


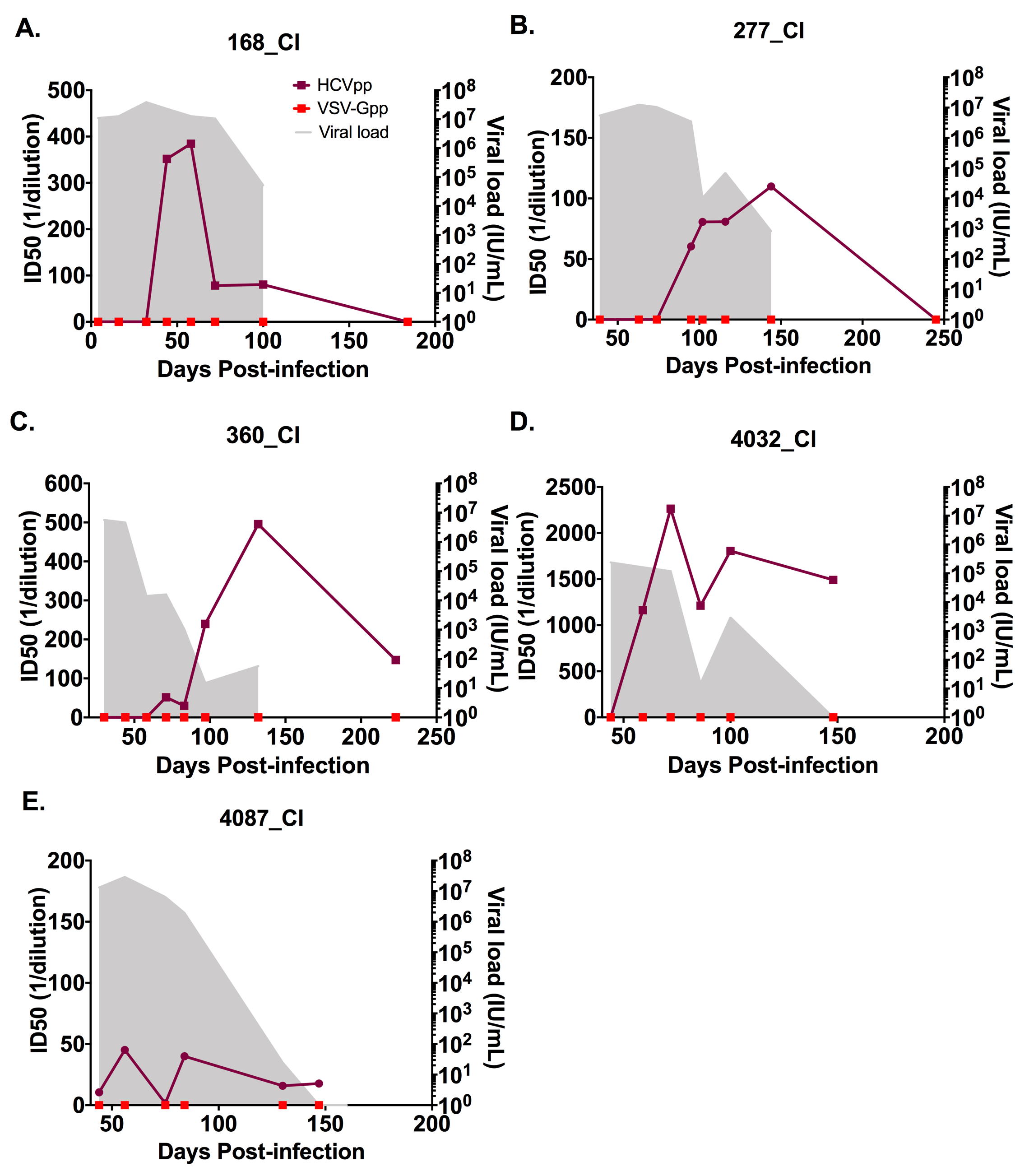

Figure S 3 HCV and VSV-G neutralizing antibody (nAb) responses and HCV RNA levels (IU/ml) examined longitudinally for clearers and chronic progressors. Panels A - E shows subjects who cleared the infection. Panels F - J shows subjects who developed chronic HCV infection. The shaded area represents the longitudinal HCV RNA levels (IU/ml). The maroon line represents HCV nAb ID50 titer with squares representing timepoints tested on autologous virus and circles representing timepoints tested on heterologous virus. The red line represents neutralization of control pseudo-particle VSV-G. All neutralization results were generated from quadruplicates using two-fold dilutions from 1/40 to 1/2560.

Figure S 4 HCV neutralizing antibody (nAb) responses examined longitudinally for clearers and chronic progressors. Each panel represents one subject and each coloured symbol represents a unique longitudinal sample, classified by days post-infection. Panel A-G shows subject who cleared the infection. Panel H-N shows subjects who developed chronic HCV infection. Neutralization was performed in 2 fold dilutions for each timepoint. The error bars indicate the standard error calculated for each time point which was performed in quadruplicate. The ID50 was calculated for each time point from this data.

Figure S 5 Neutralisation breadth of clearer and chronic progressors. NAb breadth was calculated longitudinally for both clearer and chronic progressors. Samples in early acute phase of infection for chronic progressors and samples at the last RNA positive time point for clearers were tested. All samples were tested on E1E2 expressing subtypes 1a, 1b, 2a, 2b, 3a, 4a and 6a. Breadth is represented in a heat map, illustrating neutralization results (at 1/50) against heterologous HCVpp for clearers and chronic progressors.

**Figure S6. Screening of longitudinal plasma against the dominant viral variants that emerge over the course of infection for two chronic progressors,** THD_Ch and THG_Ch. Both subjects were infected by the same T/F variant and developed different longitudinal variants that were dominant at the days post-infection indicated by the number in the figure legend. In both subjects nAb activity towards the T/F variant was delayed, but emerging neutralization activity could be detected slightly earlier towards the longitudinal variants but this was still below the 50% cutoff for defining the presence of nAb activity. This does suggest that nAb activity is truly delayed to the T/F variant and likely develops after T/F is cleared and towards a longitudinal variant and then as the response broadens is cross-reactive to the T/F variant as the response broadens. THG_T/F and THG_58 had identical data and are overlayed on the figure.

Figure S 7 Longitudinal non-autologous competition based epitopes mapping in clearer and chronic progressors. Nine monoclonal antibodies (mAbs) were used to determine which epitopes were targeted throughout infection (see key). The maroon arrow indicates first nAb response (see key) and grey shading represents viral load (see key). Panels A- E show subjects who developed chronicity and panels F – H show subjects who cleared infection. Subjects 4032_Cl, 686_Cl and 360_Cl are not shown as samples either could not be mapped or showed no competition.


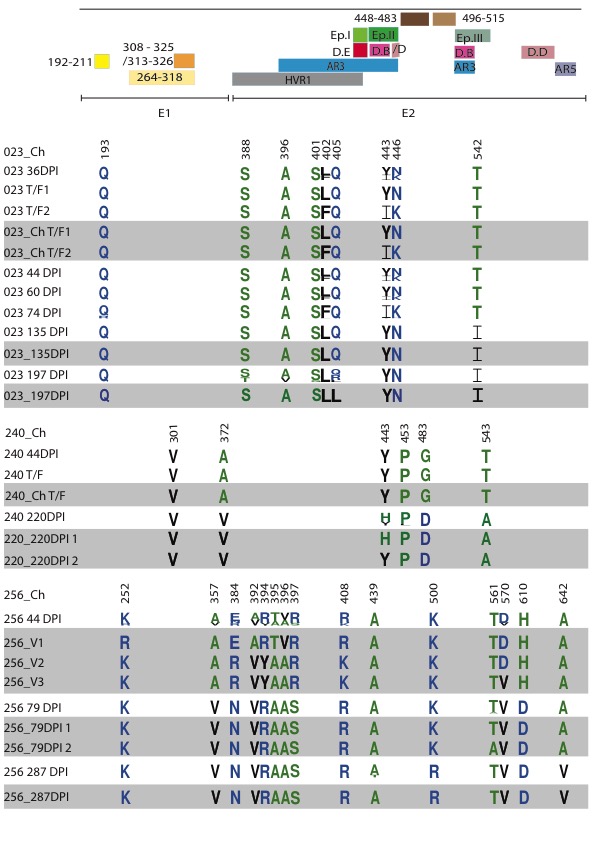


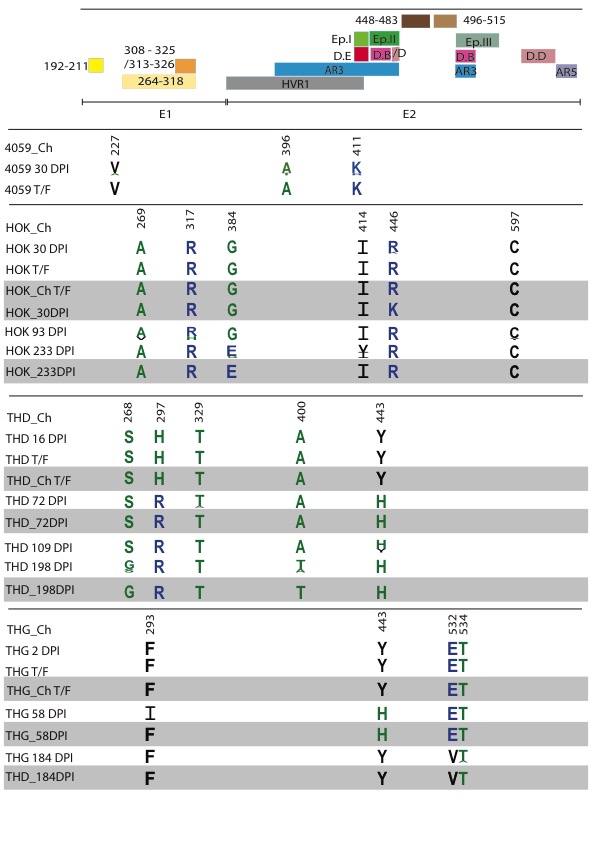


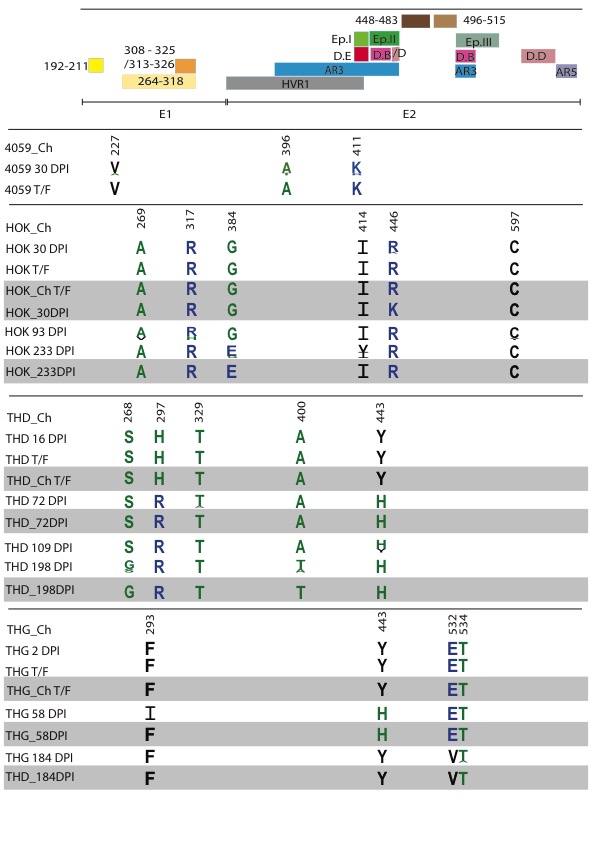


Figure S 8 Longitudinal amino acid changes in E1E2 in clearers and chronic progressors. Samples of 6 subjects with chronic HCV were subjected to NGS and upon analysis cloned over the E1E2 region. The variant frequency of amino acids as determined by NGS analysis is shown longitudinally for subjects (n = 6) and timepoints where SAPs occurred at over 10% of the population. Amino acid differences in E1E2 are shown with reference to the earliest time point available for each subject. Symbol height is proportional to amino acid frequency per site. Clones generated are positioned under the corresponding time point and highlighted in grey. NAb epitopes and variable regions are labelled. Those occurring in E1 are represented in yellow, HVR1 is represented in grey, regions 448-483 and 496-515 in brown, AR1-5 in blue, Domains (D) A-E in red and Epitopes (E) 1-III in green. Visualisation was produced from WebLogo.berkely.edu. Residue numbering is in reference to H77

**Figure S9. Cross-competition matrix for the mAbs used for epitope mapping.** Cross competition immunoassays were performed for all E1E2-specific antibodies that were used to characterise patient anti HCV E1E2 Ab responses. First column lists the antibodies used to compete and the first row indicates the biotinylated antibody used for detection.

Table S 1 Monoclonal antibody binding to pseudo-typed transmitted/founder and longitudinal variants using an ELISA based method

| **E1E2 glycoprotein** | **Monoclonal Antibody** | | | | | | | | | | | |
| --- | --- | --- | --- | --- | --- | --- | --- | --- | --- | --- | --- | --- |
|  | **AR1B** | **AR2A** | **AR3A** | **AR4A** | **AR5A** | **HCV84.26** | **CBH4G** | **CBH5** | **CBH7** | **HCV1** | **A8** | **MAb24** |
| 4087_Cl | 15.28 | 3.89 | 18.61 | 13.19 | 16.53 | 17.47 | 2.02 | 18.32 | 12.18 | 8.40 | 20.22 | 0.00 |
| 306_Cl | 0.00 | 4.00 | 8.71 | 0.00 | 0.00 | 7.66 | 9.40 | 9.00 | 14.57 | 7.56 | 12.31 | 0.00 |
| 277_Cl | 0.00 | 0.00 | 0.00 | 0.00 | 0.00 | 0.00 | 0.00 | 0.00 | 0.00 | 0.00 | 0.00 | 0.00 |
| 306_Cl | 0.00 | 0.00 | 19.07 | 16.39 | 18.41 | 0.00 | 6.97 | 5.83 | 0.00 | 8.37 | 19.14 | 0.00 |
| 4032_Cl | 0.00 | 0.00 | 3.30 | 0.00 | 2.20 | 2.09 | 0.00 | 3.97 | 3.17 | 2.62 | 4.59 | 0.00 |
| 168_Cl | 18.33 | 4.16 | 8.07 | 7.01 | 14.97 | 18.45 | 5.49 | 18.57 | 17.32 | 21.27 | 19.83 | 13.51 |
| 686_Cl | 16.51 | 3.27 | 0.00 | 15.48 | 19.14 | 0.00 | 3.15 | 4.25 | 16.65 | 17.96 | 9.10 | 5.90 |
| 023_T/F1 | 18.51 | 15.14 | 16.45 | 5.81 | 14.77 | 6.80 | 6.49 | 16.03 | 18.34 | 19.26 | 18.32 | 4.99 |
| 023_T/F2 | 15.18 | 8.82 | 9.27 | 2.20 | 6.33 | 0.00 | 4.18 | 5.86 | 17.53 | 10.94 | 0.00 | 0.00 |
| 023_135DPI | 2.48 | 0.00 | 2.09 | 0.00 | 0.00 | 0.00 | 0.00 | 2.00 | 5.08 | 3.38 | 2.32 | 0.00 |
| 023_197DPI | 3.45 | 0.00 | 2.49 | 0.00 | 2.92 | 0.00 | 0.00 | 2.55 | 6.78 | 4.50 | 2.82 | 0.00 |
| 240_Ch T/F | 0.00 | 0.00 | 3.68 | 0.00 | 3.86 | 3.50 | 0.00 | 3.10 | 3.87 | 4.35 | 4.14 | 0.00 |
| 240_220DPI 1 | 0.00 | 0.00 | 3.27 | 0.00 | 3.43 | 3.06 | 0.00 | 2.72 | 3.04 | 2.59 | 3.94 | 0.00 |
| 240_220DPI 2 | 0.00 | 0.00 | 3.90 | 0.00 | 4.11 | 3.28 | 0.00 | 2.82 | 3.80 | 3.28 | 4.47 | 0.00 |
| THD_Ch T/F | 18.31 | 6.72 | 0.00 | 9.80 | 19.12 | 6.63 | 0.00 | 4.94 | 17.92 | 14.08 | 14.71 | 3.30 |
| THD_72 DPI | 19.16 | 10.91 | 0.00 | 16.14 | 20.74 | 3.02 | 2.26 | 3.44 | 18.64 | 14.91 | 11.54 | 3.26 |
| THD_198 DPI | 15.17 | 5.25 | 0.00 | 9.85 | 18.53 | 2.04 | 0.00 | 2.14 | 16.03 | 9.56 | 9.12 | 0.00 |
| THG_Ch T/F | 18.31 | 6.72 | 0.00 | 9.80 | 19.12 | 6.63 | 0.00 | 4.94 | 17.92 | 14.08 | 14.71 | 3.30 |
| THG_58 DPI | 18.09 | 11.65 | 2.36 | 15.19 | 21.29 | 3.18 | 0.00 | 4.34 | 18.25 | 18.02 | 15.00 | 4.41 |
| THG_184 DPI | 18.10 | 10.26 | 2.93 | 12.04 | 20.79 | 9.78 | 0.00 | 0.00 | 18.51 | 19.84 | 18.66 | 6.26 |
| HOK_Ch 30 DPI | 13.91 | 0.00 | 15.43 | 12.37 | 15.54 | 13.38 | 2.62 | 15.58 | 12.09 | 6.29 | 18.05 | 2.11 |
| HOK_Ch T/F | 0.00 | 0.00 | 0.00 | 0.00 | 0.00 | 0.00 | 0.00 | 0.00 | 0.00 | 0.00 | 0.00 | 0.00 |
| HOK_233 DPI | 0.00 | 0.00 | 0.00 | 0.00 | 0.00 | 0.00 | 0.00 | 2.24 | 2.85 | 0.00 | 2.21 | 0.00 |
| 256_Ch V1 | 17.56 | 10.73 | 13.62 | 12.55 | 21.14 | 12.70 | 2.12 | 16.16 | 18.96 | 17.30 | 14.74 | 5.18 |
| 256_Ch V2 | 15.95 | 8.76 | 6.27 | 10.96 | 18.78 | 10.25 | 2.33 | 11.64 | 18.62 | 9.46 | 10.38 | 2.67 |
| 256_Ch V3 | 8.23 | 3.57 | 5.30 | 4.74 | 10.42 | 5.38 | 0.00 | 7.65 | 14.90 | 8.12 | 6.76 | 0.00 |
| 256_79 DPI 1 | 12.25 | 9.84 | 6.01 | 4.34 | 17.42 | 6.48 | 0.00 | 9.99 | 17.43 | 11.65 | 8.48 | 2.67 |
| 256_79 DPI 2 | 2.08 | 2.20 | 0.00 | 0.00 | 3.17 | 0.00 | 0.00 | 2.21 | 3.94 | 2.89 | 2.08 | 0.00 |
| 256_287 DPI | 14.52 | 16.08 | 8.76 | 7.79 | 18.48 | 10.00 | 0.00 | 12.76 | 18.49 | 12.90 | 11.75 | 2.97 |
| Monoclonal antibody binding ability is represented by signal (HCVpp binding OD) over noise (negative control binding OD (pseudo-particle generated without Envelope glycoproteins). | | | | | | | | | | | | |
|  |  |  |  |  |  |  |  |  |  |  |  |  |

Table S 2 Clones used for non-autologous neutralisation

| **Subject** | **HCVpp^a^ clone** | **Pairwise distance (%)** |
| --- | --- | --- |
| 277_Cl | UKN3A13.6 | 6 |
| 306_Cl | UKN2B2.8 | 9 |
| 4087_Cl | UKN1B5.23 | 7 |
| 240_Ch | UKN3A1.28 | 7 |
| 4059_Ch | UKN2B2.8 | 8 |

^a^ HCV pseudo-particle

| Table S 3 Subjects with SAPs occurring in each epitope   \| **Epitope** \| **Clearer (7^a^)** \| **Chronic (7 ^b^)** \| **p value** \| \| --- \| --- \| --- \| --- \| \| AR5 \| 1 \| 1 \| >0.9999 \| \| AR4 \| 1 \| 0 \| >0.9999 \| \| **AR3** \| **2** \| **7** \| **0.041** \| \| Epitope I \| 0 \| 1 \| >0.9999 \| \| **Epitope II** \| **1** \| **6** \| **0.029** \| \| Epitope III \| 0 \| 3 \| 0.282 \| \| **Domain B** \| **0** \| **5** \| **0.041** \| \| Domain E \| 0 \| 1 \| >0.9999 \| \| Domain D \| 2 \| 5 \| 0.286 \| \| HVR1 \| 2 \| 5 \| 0.286 \| \| E2 448-483 \| 1 \| 2 \| >0.9999 \| \| E2 496-515 \| 1 \| 1 \| >0.9999 \| \| E1 192-211 \| 0 \| 1 \| >0.9999 \| \| E1 264-318 \| 1 \| 4 \| >0.9999 \| \| E1 308-325 \| 1 \| 1 \| >0.9999 \| \| E1 313-327 \| 0 \| 1 \| >0.9999 \| \| ^a^ Total number of clearers (n = 7), ^b^ Total number of chronic progressors (n = 7) \| \| \| \| |
| --- | --- | --- | --- | --- | --- | --- | --- | --- | --- | --- | --- | --- | --- | --- | --- | --- | --- | --- | --- | --- | --- | --- | --- | --- | --- | --- | --- | --- | --- | --- | --- | --- | --- | --- | --- | --- | --- | --- | --- | --- | --- | --- | --- | --- | --- | --- | --- | --- | --- | --- | --- | --- | --- | --- | --- | --- | --- | --- | --- | --- | --- | --- | --- | --- | --- | --- | --- | --- | --- | --- | --- | --- |
